# Absence of genetic association between insulin-like growth factors and esophageal cancer

**DOI:** 10.1097/MD.0000000000040899

**Published:** 2024-12-27

**Authors:** Zhengliang Sun, Xiaohong Wang

**Affiliations:** a Department of Thoracic Surgery, East Hospital of Tongji University, Pudong District, Shanghai, China; b Longhua Street Community Health Center, Xuhui District, Shanghai, China.

**Keywords:** esophageal cancer (ESC), IGF-binding proteins (IGFBPs), instrumental variables (IVs), insulin-like growth factors (IGFs), Mendelian randomization (MR)

## Abstract

This study aimed to explore the causal relationship between concentrations of various insulin-like growth factors (IGFs) and IGF-binding proteins (IGFBPs) and esophageal cancer (ESC), addressing the gap in understanding the genetic link between IGF1 and ESC. A two-sample Mendelian randomization (MR) analysis was conducted using single nucleotide polymorphisms linked to IGFs/IGFBPs and ESC from the IEU Open GWAS Project. This analysis included ESC GWAS data from 1996 individuals of European descent and genetic variant data from 3310 individuals of European ancestry. Various methods, such as inverse variance weighting, weighted median, weighted mode, and MR-Egger regression, were applied for analysis, with sensitivity assessments including MR-PRESSO, Cochran Q, and leave-one-out analysis to ensure the robustness of results and detect biases. The genetic predictions indicated no significant association between IGFs/IGFBPs and ESC. When ESC was the outcome measure, the odds ratios with 95% confidence intervals were as follows: IGF1 = 1.00 (0.89–1.12, *P* = .936), IGF1R = 1.07 (0.90–1.27, *P* = .453), IGFBP3 = 1.00 (0.79–1.26, *P* = .975), and IGFBPL1 = 0.91 (0.75–1.12, *P* = .372). MR-Egger regression confirmed the absence of horizontal pleiotropy, and no outliers were identified by MR-PRESSO. Leave-one-out analysis supported the stability of the results. The study did not find a causal connection between IGFs/IGFBPs and ESC. These results suggest the need for further validation and potentially highlight the complex interplay of factors involved in the development of ESC.

## 1. Introduction

Esophageal cancer (ESC) is the 8th most common malignancy and the 6th leading cause of cancer-related deaths worldwide, with 604,100 new cases and 544,076 deaths projected in 2020.^[1]^ Esophageal adenocarcinoma is rapidly evolving and represents the predominant subtype of ESC among Caucasians in Europe, North America, and Australia.^[2]^ Barrett esophagus is a common precursor lesion of esophageal adenocarcinoma, caused by the recurrent proliferation of esophageal cells due to long-term gastroesophageal reflux disease.^[3]^ The initial symptoms of ESC are often subtle and easily overlooked, leading to most patients being diagnosed at an advanced stage, which can account for up to 90% of cases. Consequently, these patients often miss the optimal window for diagnosis and treatment, resulting in decreased quality of life, increased mortality, poor prognosis, and a 5-year survival rate of <20%,^[4]^ thereby creating a significant disease burden. Primary prevention of cancer can be achieved by investigating the common influencing factors associated with ESC, its precancerous lesions, and related diseases, alongside implementing appropriate attention and intervention measures.

Epidemiological studies have identified sex steroids, insulin-like growth factors (IGFs), and adipokines as the 3 primary models explaining the biological basis of the relationship between obesity and cancer, including ESC.^[5]^ IGFs are important growth-promoting peptides that act through the IGF1 receptor.^[6,7]^ IGF-I and IGF-II are primarily produced by the liver and circulate in the bloodstream, but they are also locally produced in tissues, exerting effects in a paracrine or autocrine manner.^[7]^ IGF-I regulates cellular growth, differentiation, and survival both through systemic circulation and within tissues. Most of these growth factors circulate bound to IGF-binding proteins (IGFBPs), which extend the half-life of IGFs and regulate IGF signaling.^[6,8]^ Elevated IGF-I signaling enhances cell survival and reduces apoptosis, thereby increasing the likelihood of carcinogenesis.^[8,9]^ Furthermore, circulating concentrations of IGF-I have been positively correlated with the risk of developing several cancers, including esophageal, prostate, and breast cancers.^[10,11]^ IGFBP3 has been reported to inhibit the proliferation and metastasis of ESC and is negatively associated with the risk of ESC.^[12,13]^ Clinical studies have demonstrated that IGF1R is highly expressed in various human tumors, including ESC, and is associated with reduced overall survival in adenocarcinoma, but not in squamous cell carcinoma.^[14,15]^ Notably, these observational studies may be limited by sample size and potential confounding factors; thus, the potential causal relationship between genetic susceptibility to ESC and IGF risk remains unclear.

Mendelian randomization (MR) is a novel method based on Mendel first and second laws of genetic inheritance: the law of segregation and the law of independent assortment.^[16]^ A genetic variant can be regarded as an instrumental variable (IV) for a given exposure if it satisfies the IV assumptions: (a) it does not affect the outcome; (b) it is not associated with the outcome through confounding pathways; and (c) it is associated with the exposure.^[17]^ Several MR studies have been conducted to further elucidate risk factors related to respiratory diseases. For example, Zhang et al used MR and found a negative causal relationship between educational attainment and ESC.^[18]^ Gao et al reported a causal relationship between gut microbiota and ESC, identifying 11 types of gut microbiota associated with the disease.^[19]^ However, the relationship between IGF and ESC using the MR method has not yet been investigated.

In this study, we applied an MR analysis to investigate the causal association between IGF, encompassing both IGFs and IGFBPs, and ESC.

## 2. Methods

### 2.1. Study design

**Figure 1.**
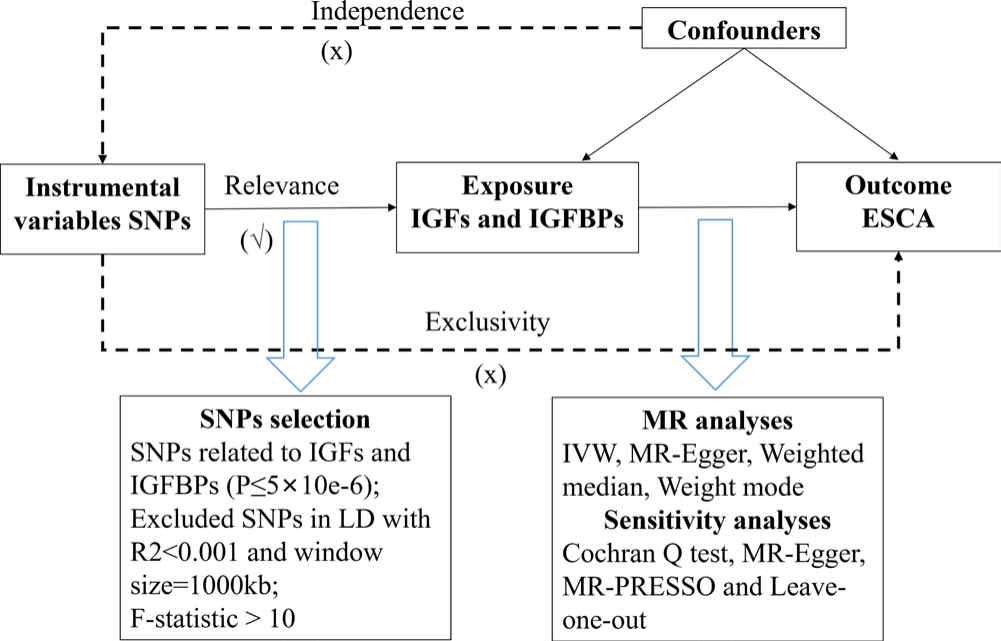
Overview of Mendelian randomization.

The MR analysis adhered to the Strengthening the Reporting of Observational Studies in Epidemiology using Mendelian Randomization (STROBE-MR) guidelines.^[20]^ The analysis followed 3 assumptions of MR studies^[17]^ (Fig. 1). Ethical approval was not required for our analysis, as it relied on published studies and publicly available GWAS summary data, without the need for original data collection.

### 2.2. Data source

To examine the unbiased causal connection between the occurrence of ESC and IGF, a two-sample MR method was employed in this research. The abundance of IGF served as the exposure factor, while the occurrence of ESC was considered the outcome factor. The GWAS summary dataset of genetic variants associated with ESC was obtained from the public database IEU Open GWAS and comprised 998 cases and 998 controls, encompassing 24,194,380 single nucleotide polymorphisms (SNPs) of European ancestry.^[21]^ IGF-related GWAS data, which included IGF1R, IGF1, IGFBP1, IGFBP2, IGFBP3, IGFBP6, IGFBP7, and IGFBPL1, were sourced from literature datasets included in IEU Open GWAS, comprising 3301 samples and 10,534,735 SNPs of European ancestry^[22]^ (Table 1).

**Table 1:** The GWAS data for exposure and outcomes.

Trait	GWAS ID	Population		SNPs
		Case/control	Decent	
ESC	ebi-a-GCST90018841	998/998	European	24,194,380
IGF1R	prot-a-1444	3301	European	10,534,735
IGF1	prot-a-1443	3301	European	10,534,735
IGFBP11	prot-a-1446	3301	European	10,534,735
IGFBP2	prot-a-1448	3301	European	10,534,735
IGFBP3	prot-a-1449	3301	European	10,534,735
IGFBP6	prot-a-1450	3301	European	10,534,735
IGFBP7	prot-a-1451	3301	European	10,534,735
IGFBPL1	prot-a-1452	3301	European	10,534,735

ESC = esophageal cancer, IGF1 = insulin-like growth factor 1, IGF1R = insulin-like growth factor 1 receptor, IGFBP1 = insulin-like growth factor-binding protein 1, IGFBP2 = insulin-like growth factor-binding protein 2, IGFBP3 = insulin-like growth factor-binding protein 3, IGFBP6 = insulin-like growth factor-binding protein 6, IGFBP7 = insulin-like growth factor-binding protein 7, IGFBPL1 = insulin-like growth factor-binding protein-like 1.

### 2.3. IV selection

In MR studies, SNPs are used as IVs. The IVs included in this study met the following criteria: first, SNPs significantly associated with exposure in the entire genome were screened using a significance threshold of *P* < 5 × 10^-6^.^[23]^ Subsequently, SNPs with a minimum minor allele frequency >0.01 were selected.^[24]^ Linkage disequilibrium between SNPs was then minimized using the criteria *R*² < 0.001 and a window size of 10,000 kb.^[25]^ When selected IVs were absent from the outcome summary data, SNPs with high linkage disequilibrium (*R*² > 0.8) were searched as proxy SNPs to replace the existing ones.^[26]^ The F-value for each SNP in the IV was calculated to assess IV strength, excluding potential weak instrument bias between the IV and exposure factors, using the formula: F *=* *R*^2^ × (N - 2)/(1 - *R*^2^), where *R*² represents the proportion of exposure variance explained by the SNP in the IV. The requirement for the F-value was > 10.^[27]^

### 2.4. MR analysis

The inverse variance weighting (IVW) method was utilized to assess the causal association between IGF and ESC by calculating the odds ratio (OR) and 95% confidence interval (CI).^[28]^ The MR-Egger, weighted median (WM), and weighted mode methods were employed to test the robustness of the results.^[29]^ The MR-Egger method accounts for the presence of intercept and provides accurate causal effect estimates in the presence of pleiotropic bias; if pleiotropic effects do not cancel out (*P*-pleiotropy < .05), the IVW estimate exhibits a marked bias.^[28,30]^ The WM method assumes that half of the IVs are valid, analyzing the causal association between exposure and outcome.^[31]^ All analyses in this study were conducted using the “Two Sample MR” package in R version 4.3.2. MR results were visualized using scatter plots and forest plots.

### 2.5. Sensitivity analysis

A sensitivity analysis was conducted to detect potential heterogeneity and horizontal pleiotropy in the MR studies. Cochran *Q* test was employed to assess heterogeneity among IVs, with *P* > .05 indicating low heterogeneity. This suggests that the estimates among IVs were randomly distributed and had little impact on the IVW results.^[32]^ To account for the potential impact of genetic variation on the estimation of association effects, the MR-Egger regression method was used to explore the presence of horizontal pleiotropy.^[33,34]^ When the intercept of the MR-Egger regression approaches zero or is not statistically significant, this indicates the absence of pleiotropy. Additionally, the MR-PRESSO method was utilized to detect potential outliers (i.e., SNPs with *P* < .05) and to reestimate causal associations after their removal, correcting for horizontal pleiotropy.^[34]^ Funnel plots and leave-one-out plots were employed to assess the stability and consistency of the results.

## 3. Results

### 3.1. IV selection

In this study, ESC-related IVs were screened, and the specific number of SNPs, F-values, and the structure of the excluded SNPs are presented in Table 2 and Table S1, Supplemental Digital Content, http://links.lww.com/MD/O194. Additionally, when IGFBP2 was the exposure, 1 SNP (rs16856924) did not match the information in the summary data. Information on all SNPs can be found in the Data of IVs, Supplemental Digital Content, http://links.lww.com/MD/O195.

**Table 2:** F-statistical values for factor-related IVs.

Exposure	nSNP	meanF	minF	maxF	Excluded SNPs	Diff
IGF1	18	25.78401	20.83959	48.51265	rs2854746	NA
IGF1R	10	26.02243	21.52672	46.05122	rs4924807	NA
IGFBP1	9	22.25502	20.83486	25.61171	NA	NA
IGFBP2	11	22.06749	20.8717	25.27879	NA	rs16856924
IGFBP3	10	28.94228	20.97901	82.33203	rs9855615	NA
IGFBP6	13	23.66713	21.16155	29.02011	NA	NA
IGFBP7	11	35.50422	20.91958	166.4101	NA	NA
IGFBPL1	8	23.28547	21.42862	25.28063	NA	NA

### 3.2. Causal association between IGF and ESC

The results of genetic prediction revealed no statistical association between IGF and ESC. No significant associations were observed between ESC and IGF1 (OR (95% CI): 1.001 (0.89–1.12), *P* = .936), IGF1R (OR (95% CI): 1.07 (0.90–1.27), *P* = .453), IGFBP1 (OR (95% CI): 1.06 (0.92–1.22), *P* = .445), IGFBP2 (OR (95% CI): 0.92 (0.79–1.07), *P* = .261), IGFBP3 (OR (95% CI): 1.01 (0.79–1.26), *P* = .975), IGFBP6 (OR (95% CI): 1.08 (0.89–1.32), *P* = .466), IGFBP7 (OR (95% CI): 0.96 (0.82–1.12), *P* = .607), and IGFBPL1 (OR (95% CI): 0.91 (0.75–1.12), *P* = .372) (Table 3). Furthermore, the other 3 methods, including MR-Egger, WM, and weighted mode methods, also exhibited no statistical significance. The scatter plot of genetic IVs against their respective effect estimates for both the exposure and outcome revealed no discernible pattern suggestive of a causal relationship Figure S1, Supplemental Digital Content, http://links.lww.com/MD/O193. The points were dispersed without a clear directional trend, consistent with the absence of a causal link between the exposure and outcome. The forest plot showed no overall significant effect when all variants were meta-analyzed together Figure S2, Supplemental Digital Content, http://links.lww.com/MD/O193.

**Table 3:** Relationship between IGF and ESC.

Exposure	Outcome	nSNP	Methods	OR (95% CI)	*P*
IGF1	ESC	17	MR-Egger	1.11 (0.81–1.52)	.518659
		17	WM	0.97 (0.84–1.12)	.641979
		17	IVW	1 (0.89–1.12)	.935583
		17	WM	0.96 (0.79–1.16)	.669027
IGF1R		9	MR-Egger	1.57 (0.8–3.09)	.229049
		9	WM	1.09 (0.86–1.37)	.479391
		9	IVW	1.07 (0.9–1.27)	.45348
		9	WM	1.15 (0.85–1.56)	.376436
IGFBP1		9	MR-Egger	1.21 (0.88–1.66)	.288146
		9	WM	1.07 (0.88–1.29)	.498752
		9	IVW	1.06 (0.92–1.22)	.444641
		9	WM	1.08 (0.9–1.3)	.426464
IGFBP2		11	MR-Egger	1.21 (0.6–2.42)	.609685
		11	WM	0.87 (0.71–1.07)	.193029
		11	IVW	0.92 (0.79–1.07)	.260619
		11	WM	0.83 (0.66–1.06)	.159801
IGFBP3		9	MR-Egger	0.97 (0.6–1.57)	.904121
		9	WM	0.9 (0.7–1.15)	.386317
		9	IVW	1 (0.79–1.26)	.975392
		9	WM	0.86 (0.65–1.14)	.321457
IGFBP6		13	MR-Egger	1.24 (0.81–1.9)	.335908
		13	WM	1.18 (0.94–1.49)	.15088
		13	IVW	1.08 (0.89–1.32)	.446414
		13	WM	1.35 (0.99–1.85)	.085648
IGFBP7		11	MR-Egger	1.06 (0.7–1.61)	.781303
		11	WM	0.98 (0.81–1.18)	.810848
		11	IVW	0.96 (0.82–1.12)	.606875
		11	WM	0.96 (0.77–1.19)	.724473
IGFBPL1		8	MR-Egger	1.13 (0.71–1.8)	.625615
		8	WM	0.99 (0.77–1.29)	.962425
		8	IVW	0.91 (0.75–1.12)	.371621
		8	Weighted Mode	1.02 (0.75–1.4)	.902363

ESC = esophageal cancer, IGF1 = insulin-like growth factor 1, IGF1R = insulin-like growth factor 1 receptor, IGFBP1 = insulin-like growth factor-binding protein 1, IGFBP2 = insulin-like growth factor-binding protein 2, IGFBP3 = insulin-like growth factor-binding protein 3, IGFBP6 = insulin-like growth factor-binding protein 6, IGFBP7 = insulin-like growth factor-binding protein 7, IGFBPL1 = insulin-like growth factor-binding protein-like 1, IVW = inverse variance weighted, WM = weighted median.

### 3.3. Pleiotropy, heterogeneity, and sensitivity analysis

Cochran *Q* test revealed significant heterogeneity (*P* < .05) among the IGFBP6 IVs utilized in the MR analysis. Despite this indication of heterogeneity, the adoption of a random-effects model for the IVW analysis was deemed appropriate, as it accommodates variability in effect sizes across different genetic instruments. This approach acknowledges the presence of heterogeneity and provides a more conservative estimate of the overall effect, thereby preserving the validity and robustness of our conclusions regarding the relationship between IGF and ESC (Table 4). The results of the MR-Egger regression indicated that this analysis was not influenced by horizontal pleiotropy and remained robust, as shown in Table 4. Additionally, the MR-PRESSO results suggested the absence of outliers, as presented in Table 5. The funnel plot, utilized to assess publication bias and heterogeneity among studies, displayed symmetry around the summary effect estimate, indicating minimal risk of bias due to small study effects or selective reporting Figure S3, Supplemental Digital Content, http://links.lww.com/MD/O193. This symmetry, combined with the consistently nonsignificant findings, reinforces our conclusion that there is no causal relationship between IGF and ESC. To further examine the stability of our findings, we conducted a leave-one-out sensitivity analysis; the resulting plot demonstrated that the exclusion of any single variant did not materially alter the overall conclusion of no causal relationship, underscoring the consistency and robustness of our findings against potential outlier effects Figure S4, Supplemental Digital Content, http://links.lww.com/MD/O193.

**Table 4:** Horizontal pleiotropy and heterogeneity tests.

Exposure	Outcome	Heterogeneity		Pleiotropy	
		Q statistics (IVW)	*P* value	MR-Egger intercept	*P* value
IGF1	ESC	4.14	1.00	-0.03	.47
IGF1R		3.80	.87	-0.07	.28
IGFBP1		4.71	.79	-0.04	.40
IGFBP2		9.27	.51	-0.05	.45
IGFBP3		12.71	.12	0.01	.90
IGFBP6		21.52	.04	-0.03	.47
IGFBP7		11.16	.35	-0.02	.62
IGFBPL1		8.58	.28	-0.04	.36

ESC = esophageal cancer, IGF1 = insulin-like growth factor 1, IGF1R = insulin-like growth factor 1 receptor, IGFBP1 = insulin-like growth factor-binding protein 1, IGFBP2 = insulin-like growth factor-binding protein 2, IGFBP3 = insulin-like growth factor-binding protein 3, IGFBP6 = insulin-like growth factor-binding protein 6, IGFBP7 = insulin-like growth factor-binding protein 7, IGFBPL1 = Insulin-like growth factor-binding protein-like 1.

**Table 5:** MR-PRESSO results.

Exposure	Raw				Outlier corrected				Global *P*
	OR (CI%)	Low CI	Up CI	*P*	OR (CI%)	Low CI	Up CI	*P*	
IGF1	0.99	0.94	1.05	0.736346	NA	NA	NA	NA	1
IGF1R	1.07	0.95	1.19	0.296714	NA	NA	NA	NA	.896
IGFBP1	1.06	0.95	1.18	0.348157	NA	NA	NA	NA	.837
IGFBP2	0.92	0.79	1.06	0.26981	NA	NA	NA	NA	.571
IGFBP3	1.02	0.82	1.26	0.863977	NA	NA	NA	NA	.163
IGFBP6	1.08	0.89	1.32	0.461116	NA	NA	NA	NA	.049
IGFBP7	0.96	0.82	1.12	0.618055	NA	NA	NA	NA	.38
IGFBPL1	0.91	0.75	1.12	0.401292	NA	NA	NA	NA	.312

ESC = esophageal cancer, IGF1 = insulin-like growth factor 1, IGF1R = insulin-like growth factor 1 receptor, IGFBP1 = insulin-like growth factor-binding protein 1, IGFBP2 = insulin-like growth factor-binding protein 2, IGFBP3 = insulin-like growth factor-binding protein 3, IGFBP6 = insulin-like growth factor-binding protein 6, IGFBP7 = insulin-like growth factor-binding protein 7, IGFBPL1 = insulin-like growth factor-binding protein-like 1.

## 4. Discussion

This study employed random-effects IVW to provide strong evidence for a causal relationship between ESC and IGF. Despite the expected associations and the utilization of the largest available public GWAS meta-analysis data, we were unable to demonstrate a significant association between genetic instruments for ESC and IGF (including IGF1R, IGF1, IGFBP1, IGFBP2, IGFBP3, IGFBP6, IGFBP7, and IGFBPL1). Although no correlation between IGF and ESC was found, our results may help to rule out certain hypotheses and guide the rational allocation of resources to prevent unnecessary public health interventions or changes in clinical practice. To the best of our knowledge, this is the first MR study to analyze the relationship between IGF and ESC, and the robustness of the results is enhanced by the utilization of multiple MR methods and approaches, as well as the most recent and robust ESC GWAS dataset.

ESC is a leading cause of cancer-related deaths worldwide, with multiple factors contributing to its occurrence and progression.^[35]^ Although our study suggests that IGFs do not increase the risk of developing ESC, the role of IGFs in ESC risk has been studied for many years, and the relationship between the 2 remains not fully understood.

For instance, IGFBP3 is the most abundant binding partner of IGF1 in ESC.^[36]^ Clinical studies have indicated that serum levels of IGF1 and IGFBP3 are significantly elevated in patients with ESC compared to healthy subjects. Additionally, a significant correlation has been observed between IGF1 levels and the depth of infiltration and pathological stage, alongside a lower survival rate in patients exhibiting high IGF-1 expression compared to those with low expression.^[36,37]^ However, another case–control study suggested that IGFBP3 inhibits ESC proliferation and metastasis, demonstrating a negative association with the risk of ESC.^[12,13]^ Meanwhile, our findings indicate that IGF1 and IGFBP3 are not causally associated with ESC. The discrepancies between clinical study results and the lack of correlation between the 2, as determined through MR, provide further evidence that clinical studies may be influenced by unmeasured or unknown confounding factors.

Furthermore, multiple studies have shown a correlation between IGF1R and ESC. Imsumran et al found that the blockade of IGF1R inhibits the proliferation and motility of ESC cells while enhancing chemotherapy-induced apoptosis in these cells.^[38]^ Several basic and clinical studies have concluded that inhibiting the function of IGF1R is beneficial in the treatment of ESC.^[39,40]^ However, our MR results suggest that there is no significant causal association between the 2. This discrepancy may arise because clinical study samples are not entirely representative of the general population, while genetic studies encompass a wide range of genetic variants across different populations.

Lastly, other IGFBPs have been linked to ESC. One case–control study reported that IGFBPL1 is a novel oncogenic factor in human ESC, capable of inhibiting the growth of ESC cells both in vitro and in vivo.^[41]^ Kaya et al found that IGFBP7 mRNA expression was significantly lower in ESC patients compared to controls.^[42]^ Conversely, Manoj et al reported that IGFBP7 was highly expressed in the majority (91%) of ESC patients.^[43]^ Moreover, our inverse variance weighted results indicated no causal relationship between ESC and IGFBPs (including IGFBP1, IGFBP2, IGFBP6, IGFBP7, and IGFBPL1). This suggests that any observed association may be coincidental or confounded by unknown factors. Additionally, some shared protein kinase B pathways between these diseases may contribute to a link between IGFBPs and ESC.

The results of our MR study suggest that there is no causal relationship between IGF and ESC. As mentioned previously, it has been proposed that there may be some association between IGF and ESC, but earlier studies based solely on observational data have struggled to demonstrate a causal relationship at the genomic level.^[44]^ Measurement error and bias in previous clinically relevant studies represent inherent limitations of observational studies.^[45]^ Furthermore, other confounding factors affecting the relationship between IGF and ESC, such as age and comorbidities, have often been overlooked in observational research. MR studies, which utilize genetic variants as naturally occurring IVs and mimic the design of randomized controlled trials, strongly demonstrate that there is no causative relationship between IGF and ESC. Therefore, IGF cannot be considered a generalized marker for ESC, and future clinical studies should take this into account.

Several important strengths exist in this study. This is the first two-sample MR study to investigate the causality between IGF and ESC, representing the closest approximation to randomized controlled trials and allowing for random allocation based on genotype. This study design helps mitigate some limitations of conventional observational studies, including reverse causation and potential confounding factors. The large sample sizes of the included studies and the IVs robustly associated with IGF (F statistics ≥ 10) strengthen our MR analysis. The intercepts from the MR-Egger analysis suggest that all observed causal associations are not affected by directional pleiotropy. We conducted multiple sensitivity analyses to test the influence of pleiotropy on our causal estimates, and our results remained robust across these various tests. However, some limitations should also be acknowledged. All participants included in the study were of European descent; thus, further studies are necessary to confirm the applicability of our findings to other populations. Additionally, we did not stratify exposures and outcomes by age and sex. MR can only draw conclusions regarding trait connections in the populations from which the GWAS are obtained.

In conclusion, we performed an MR analysis of the potential causal relationship between IGF and ESC using multiple genetic information methods. Although the results suggest no causal relationship between IGF and ESC, we recognize that some observational cohort studies indicate a possible causal link, and our study remains clinically informative. This highlights the need for further well-designed prospective studies to minimize bias in observational research and to provide a larger pool of GWAS data for future MR analyses.

## Author contributions

**Data curation:** Zhengliang Sun, Xiaohong Wang.

**Formal analysis:** Zhengliang Sun, Xiaohong Wang.

**Writing – original draft:** Zhengliang Sun, Xiaohong Wang.

## Supplementary Material



## References

[1] Liu CQ Ma YL Qin Q. Epidemiology of esophageal cancer in 2020 and projections to 2030 and 2040. Thorac Cancer. 2023;14:3–11. 36482832 10.1111/1759-7714.14745 PMC9807450

[2] Thrift AP. Global burden and epidemiology of Barrett oesophagus and oesophageal cancer. Nat Rev Gastroenterol Hepatol. 2021;18:432–43. 33603224 10.1038/s41575-021-00419-3

[3] Iyer PG Katzka DA. Nonendoscopic detection of barrett esophagus and esophageal adenocarcinoma: recent advances and implications. Ann Intern Med. 2021;174:1006–7. 33939476 10.7326/M20-7164 PMC8292209

[4] Fang P Zhou J Liang Z. Immunotherapy resistance in esophageal cancer: Possible mechanisms and clinical implications. Front Immunol. 2022;13:975986. 36119033 10.3389/fimmu.2022.975986 PMC9478443

[5] Ackerman SE Blackburn OA Marchildon F Cohen P. Insights into the link between obesity and cancer. Curr Obes Rep. 2017;6:195–203. 28434109 10.1007/s13679-017-0263-x

[6] Allard JB Duan C. IGF-binding proteins: why do they exist and why are there so many? Front Endocrinol. 2018;9:117. 10.3389/fendo.2018.00117 PMC5900387 29686648

[7] Hakuno F Takahashi SI. IGF1 receptor signaling pathways. J Mol Endocrinol. 2018;61:T69–86. 29535161 10.1530/JME-17-0311

[8] Leroith D Holly JMP Forbes BE. Insulin-like growth factors: ligands, binding proteins, and receptors. Mol Metab. 2021;52:101245. 33962049 10.1016/j.molmet.2021.101245 PMC8513159

[9] Weroha SJ Haluska P. The insulin-like growth factor system in cancer. Endocrinol Metab Clin North Am. 2012;41:335–50, vi. 22682634 10.1016/j.ecl.2012.04.014 PMC3614012

[10] Fu H Hu D Chen J. Repair of the injured spinal cord by schwann cell transplantation. Front Neurosci. 2022;16:800513. 35250447 10.3389/fnins.2022.800513 PMC8891437

[11] Knuppel A Fensom GK Watts EL. Circulating insulin-like growth factor-I concentrations and risk of 30 cancers: prospective analyses in UK Biobank. Cancer Res. 2020;80:4014–21. 32709735 10.1158/0008-5472.CAN-20-1281

[12] Adachi Y Nojima M Mori M. Insulin-like growth factor-1, IGF binding protein-3, and the risk of esophageal cancer in a nested case-control study. World J Gastroenterol. 2017;23:3488–95. 28596684 10.3748/wjg.v23.i19.3488 PMC5442084

[13] Yang H Xu L Qian H. Correlation between insulin-like growth factor binding protein 3 and metastasis-associated gene 1 protein in esophageal squamous cell carcinoma. Mol Med Rep. 2016;13:4143–50. 27035126 10.3892/mmr.2016.5046 PMC4838119

[14] Cao HY Guo XF Zhu XF Li S-S Zhen Y-S. A ligand-based and enediyne-energized bispecific fusion protein targeting epidermal growth factor receptor and insulin-like growth factor-1 receptor shows potent antitumor efficacy against esophageal cancer. Oncol Rep. 2017;37:3329–40. 28498434 10.3892/or.2017.5606

[15] Kalinina T Bockhorn M Kaifi JT. Insulin-like growth factor-1 receptor as a novel prognostic marker and its implication as a cotarget in the treatment of human adenocarcinoma of the esophagus. Int J Cancer. 2010;127:1931–40. 20104520 10.1002/ijc.25196

[16] Birney E. Mendelian randomization. Cold Spring Harb Perspect Med. 2022;12:a041302. 34872952 10.1101/cshperspect.a041302 PMC9121891

[17] Martens EP Pestman WR De Boer A Belitser SV Klungel OH. Instrumental variables: application and limitations. Epidemiology. 2006;17:260–7. 16617274 10.1097/01.ede.0000215160.88317.cb

[18] Zhang X Yang X Zhang T Yin X Man J Lu M. Association of educational attainment with esophageal cancer, Barrett’s esophagus, and gastroesophageal reflux disease, and the mediating role of modifiable risk factors: a Mendelian randomization study. Front Public Health. 2023;11:1022367. 37056646 10.3389/fpubh.2023.1022367 PMC10086429

[19] Gao X Wang Z Liu B Cheng Y. Causal association of gut microbiota and esophageal cancer: a Mendelian randomization study. Front Microbiol. 2023;14:1286598. 38107856 10.3389/fmicb.2023.1286598 PMC10722290

[20] Skrivankova VW Richmond RC Woolf BAR. Strengthening the reporting of observational studies in epidemiology using mendelian randomisation (STROBE-MR): explanation and elaboration. BMJ. 2021;375:n2233. 34702754 10.1136/bmj.n2233 PMC8546498

[21] Sakaue S Kanai M Tanigawa Y. A cross-population atlas of genetic associations for 220 human phenotypes. Nat Genet. 2021;53:1415–24. 34594039 10.1038/s41588-021-00931-x PMC12208603

[22] Sun BB Maranville JC Peters JE. Genomic atlas of the human plasma proteome. Nature. 2018;558:73–9. 29875488 10.1038/s41586-018-0175-2 PMC6697541

[23] Chen H Zhang Y Li S. The association between genetically predicted systemic inflammatory regulators and polycystic ovary syndrome: a mendelian randomization study. Front Endocrinol. 2021;12:731569. 10.3389/fendo.2021.731569 PMC8503255 34646235

[24] Kim JY Song M Kim MS. An atlas of associations between 14 micronutrients and 22 cancer outcomes: Mendelian randomization analyses. BMC Med. 2023;21:316. 37605270 10.1186/s12916-023-03018-y PMC10441703

[25] Clarke L Zheng-Bradley X Smith R. The 1000 Genomes Project: data management and community access. Nat Methods. 2012;9:459–62. 22543379 10.1038/nmeth.1974 PMC3340611

[26] Wan B Lu L Lv C. Mendelian randomization study on the causal relationship between leukocyte telomere length and prostate cancer. PLoS One. 2023;18:e0286219. 37352282 10.1371/journal.pone.0286219 PMC10289467

[27] Palmer TM Lawlor DA Harbord RM. Using multiple genetic variants as instrumental variables for modifiable risk factors. Stat Methods Med Res. 2012;21:223–42. 21216802 10.1177/0962280210394459 PMC3917707

[28] Bowden J Davey Smith G Haycock PC Burgess S. Consistent estimation in Mendelian randomization with some invalid instruments using a weighted median estimator. Genet Epidemiol. 2016;40:304–14. 27061298 10.1002/gepi.21965 PMC4849733

[29] Minelli C Del Greco MF Van Der Plaat DA. The use of two-sample methods for Mendelian randomization analyses on single large datasets. Int J Epidemiol. 2021;50:1651–9. 33899104 10.1093/ije/dyab084 PMC8580269

[30] Burgess S Thompson SG. Interpreting findings from Mendelian randomization using the MR-Egger method. Eur J Epidemiol. 2017;32:377–89. 28527048 10.1007/s10654-017-0255-x PMC5506233

[31] Burgess S Foley CN Allara E Staley JR Howson JMM. A robust and efficient method for Mendelian randomization with hundreds of genetic variants. Nat Commun. 2020;11:376. 31953392 10.1038/s41467-019-14156-4 PMC6969055

[32] Kulinskaya E Dollinger MB. An accurate test for homogeneity of odds ratios based on Cochran’s Q-statistic. BMC Med Res Methodol. 2015;15:49. 26054650 10.1186/s12874-015-0034-x PMC4531442

[33] Bowden J Del Greco MF Minelli C. Assessing the suitability of summary data for two-sample Mendelian randomization analyses using MR-Egger regression: the role of the I2 statistic. Int J Epidemiol. 2016;45:1961–74. 27616674 10.1093/ije/dyw220 PMC5446088

[34] Verbanck M Chen CY Neale B Do R. Detection of widespread horizontal pleiotropy in causal relationships inferred from Mendelian randomization between complex traits and diseases. Nat Genet. 2018;50:693–8. 29686387 10.1038/s41588-018-0099-7 PMC6083837

[35] Pech O. Esophageal disease. Endoscopy. 2013;45:729–33. 23990483 10.1055/s-0033-1344624

[36] Sohda M Kato H Miyazaki T. The role of insulin-like growth factor 1 and insulin-like growth factor binding protein 3 in human esophageal cancer. Anticancer Res. 2004;24:3029–34. 15517912

[37] Luo Y Hong CQ Huang BL. Serum insulin-like growth factor binding protein-3 as a potential biomarker for diagnosis and prognosis of oesophageal squamous cell carcinoma. Ann Med. 2022;54:2153–66. 35930383 10.1080/07853890.2022.2104921 PMC9359171

[38] Imsumran A Adachi Y Yamamoto H. Insulin-like growth factor-I receptor as a marker for prognosis and a therapeutic target in human esophageal squamous cell carcinoma. Carcinogenesis. 2007;28:947–56. 17183068 10.1093/carcin/bgl247

[39] Liu YC Leu CM Wong FH. Autocrine stimulation by insulin-like growth factor I is involved in the growth, tumorigenicity and chemoresistance of human esophageal carcinoma cells. J Biomed Sci. 2002;9(6 Pt 2):665–74. 12432233 10.1159/000067282

[40] Adachi Y Ohashi H Imsumran A. The effect of IGF-I receptor blockade for human esophageal squamous cell carcinoma and adenocarcinoma. Tumour Biol. 2014;35:973–85. 24026884 10.1007/s13277-013-1131-2

[41] Liu Y Zhang M He T. Epigenetic silencing of IGFBPL1 promotes esophageal cancer growth by activating PI3K-AKT signaling. Clin Epigenetics. 2020;12:22. 32041673 10.1186/s13148-020-0815-x PMC7011530

[42] Kaya Z Almali N Sahin ES Duran S Görgisen G Ates C. Association of insulin-like growth factor binding protein-7 promoter methylation with esophageal cancer in peripheral blood. Mol Biol Rep. 2022;49:3423–31. 35076852 10.1007/s11033-022-07173-y

[43] Kashyap MK Pawar HA Keerthikumar S. Evaluation of protein expression pattern of stanniocalcin 2, insulin-like growth factor-binding protein 7, inhibin beta A and four and a half LIM domains 1 in esophageal squamous cell carcinoma. Cancer Biomark. 2012;12:1–9. 23321464 10.3233/CBM-120289 PMC13016366

[44] Smith GD Ebrahim S. “Mendelian randomization”: can genetic epidemiology contribute to understanding environmental determinants of disease? Int J Epidemiol. 2003;32:1–22. 12689998 10.1093/ije/dyg070

[45] Boyko EJ. Observational research – opportunities and limitations. J Diabetes Complications. 2013;27:642–8. 24055326 10.1016/j.jdiacomp.2013.07.007 PMC3818421

