# Supplementary material for: Absence of genetic association between insulin-like growth factors and esophageal cancer
Source: Medicine (Baltimore). 2024 Dec 27;103(52):e40899. doi: 10.1097/MD.0000000000040899 (PMC11688069; doi:10.1097/MD.0000000000040899)
Supplement: Supplementary file 1 [file medi-103-e40899-s001.docx]

**Table S1.** Detailed information for all instrumental variables used in this analysis

| Trait | IGF1 | IGF1R | IGFBP1 | IGFBP2 | IGFBP3 | IGFBP6 | IGFBP7 | IGFBPL1 |
| --- | --- | --- | --- | --- | --- | --- | --- | --- |
| SNPs | rs528298 | rs75771313 | rs792048 | rs17460601 | rs73151908 | rs148789037 | rs75310357 | rs1877955 |
|  | rs12754794 | rs75180877 | rs74324167 | rs16856924 | rs9855615 | rs7603685 | rs58305168 | rs200777249 |
|  | rs147551588 | rs13060961 | rs12492993 | rs12637972 | rs145188037 | rs28879089 | rs1718849 | rs117275341 |
|  | rs35843768 | rs635634 | rs115087186 | rs7688308 | rs700746 | rs62193868 | rs9990566 | rs724167 |
|  | rs74480769 | rs7911360 | rs113894258 | rs3116833 | rs1567398 | rs79664053 | rs2076536 | rs149278241 |
|  | rs458036 | rs117074452 | rs141254998 | rs275534 | rs72735616 | rs13116176 | rs1174989 | rs12907232 |
|  | rs189861298 | rs9524857 | rs76258709 | rs2073937 | rs61866207 | rs3775298 | rs11614763 | rs133125 |
|  | rs11968607 | rs7173285 | rs12768580 | rs12242857 | rs36075284 | rs147318240 | rs12590062 | rs549204 |
|  | rs2854746 | rs59944838 | rs9897605 | rs139044263 | rs112617483 | rs62476338 | rs13335694 |  |
|  | rs17068682 | rs4924807 |  | rs7986968 | rs59074133 | rs181009192 | rs62143198 |  |
|  | rs59690153 |  |  | rs28407295 |  | rs7979870 | rs77279225 |  |
|  | rs11238719 |  |  |  |  | rs191939750 |  |  |
|  | rs117396969 |  |  |  |  | rs2247465 |  |  |
|  | rs575654 |  |  |  |  |  |  |  |
|  | rs2917521 |  |  |  |  |  |  |  |
|  | rs77575406 |  |  |  |  |  |  |  |
|  | rs10428038 |  |  |  |  |  |  |  |
|  | rs6006864 |  |  |  |  |  |  |  |

Insulin-like growth factor 1 (IGF1); Insulin-like growth factor 1 receptor (IGF1R); Insulin-like growth factor-binding protein 1 (IGFBP1); Insulin-like growth factor-binding protein 2 (IGFBP2); Insulin-like growth factor-binding protein 3 (IGFBP3); Insulin-like growth factor-binding protein 6 (IGFBP6); Insulin-like growth factor-binding protein 7 (IGFBP7); Insulin-like growth factor-binding protein-like 1 (IGFBPL1)
