# Supplementary figures and images for: Absence of genetic association between insulin-like growth factors and esophageal cancer
Source: Medicine (Baltimore). 2024 Dec 27;103(52):e40899. doi: 10.1097/MD.0000000000040899 (PMC11688069; doi:10.1097/MD.0000000000040899)

Figure S1

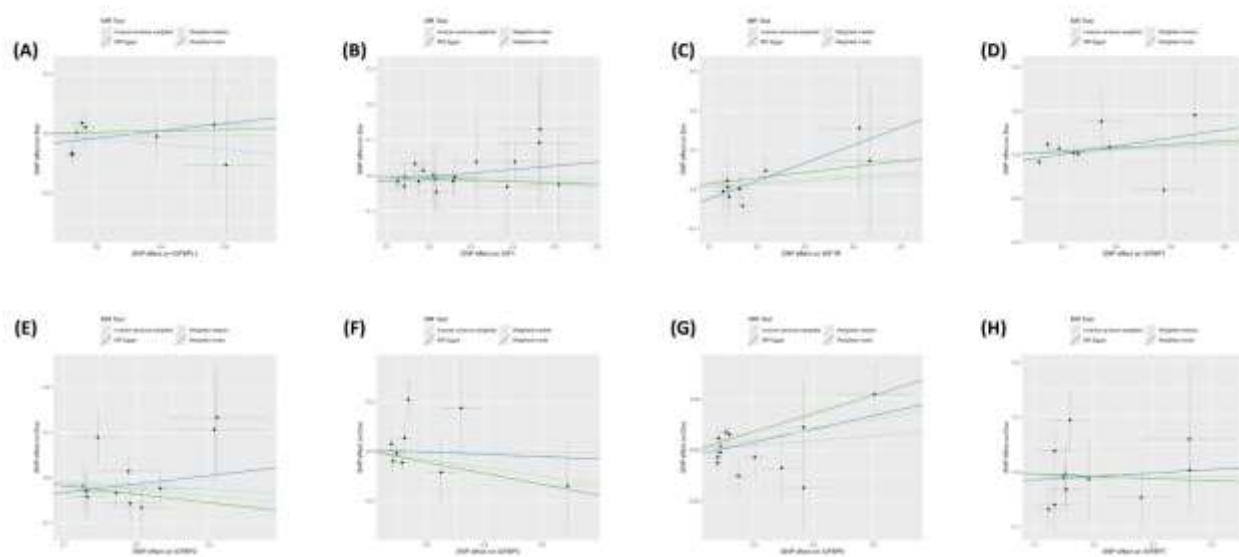

Figure S2

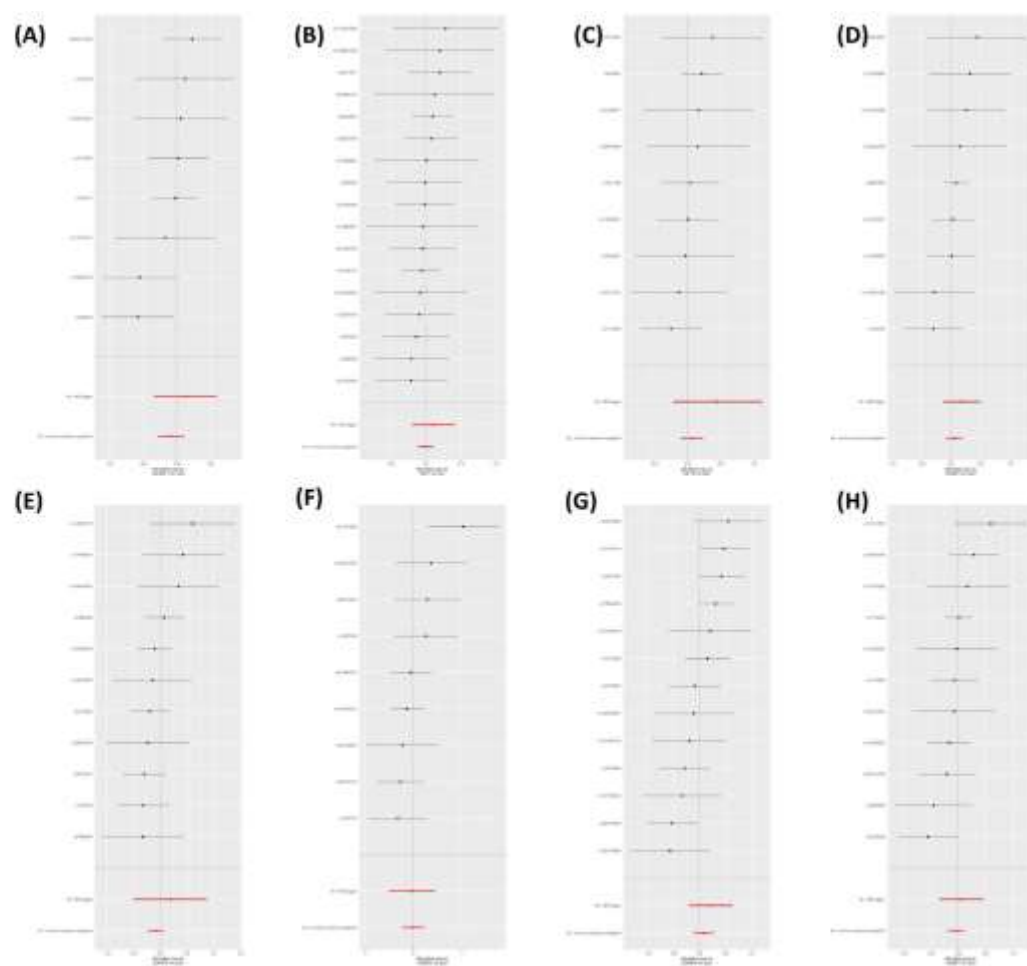

Figure S3

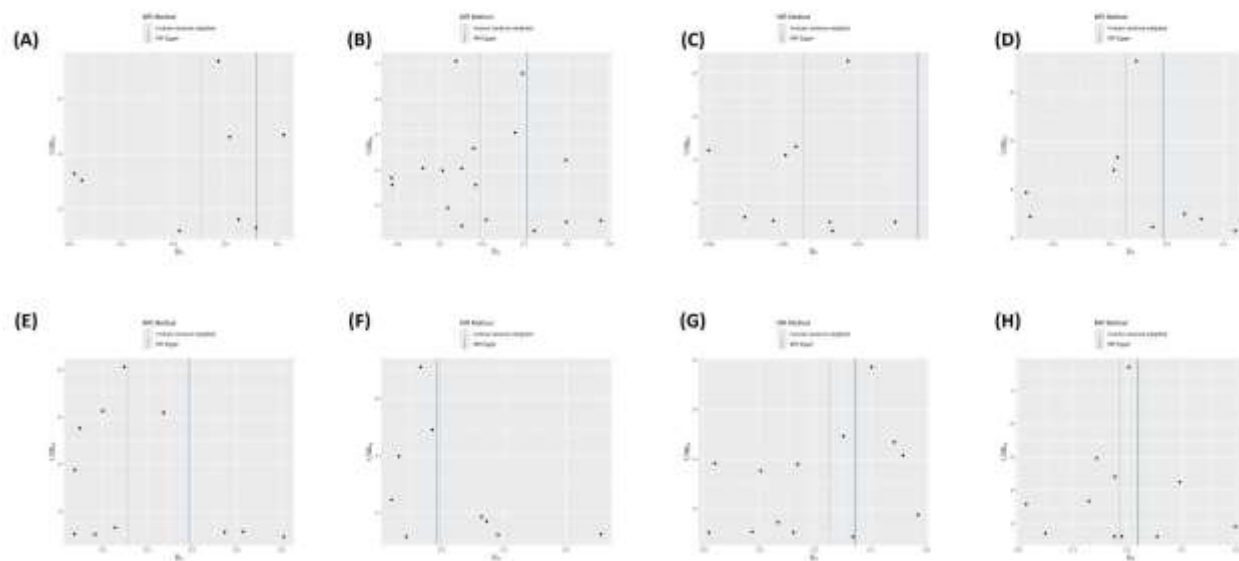

Figure S4

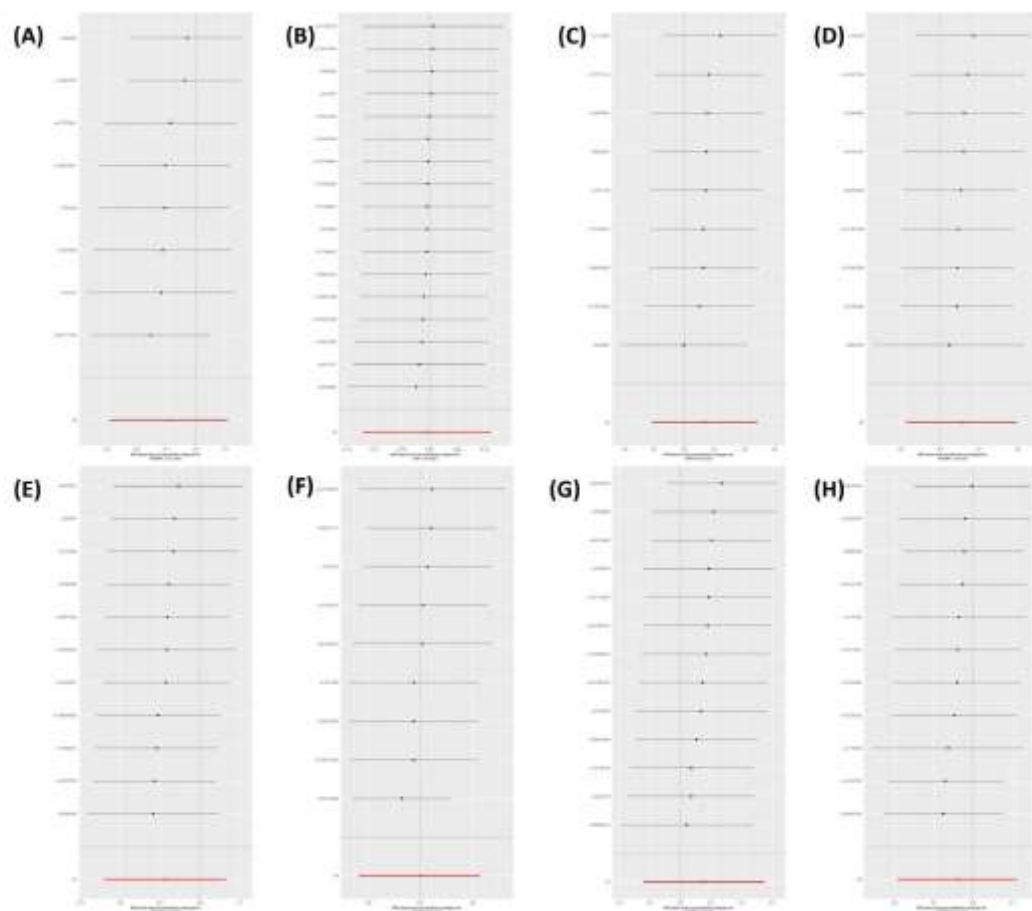

Supplement: Supplementary file 3 [file medi-103-e40899-s003.pdf]
